# Automated high-content imaging for cellular uptake, from the Schmuck cation to the latest cyclic oligochalcogenides

**DOI:** 10.3762/bjoc.16.167

**Published:** 2020-08-14

**Authors:** Rémi Martinent, Javier López-Andarias, Dimitri Moreau, Yangyang Cheng, Naomi Sakai, Stefan Matile

**Affiliations:** 1 School of Chemistry and Biochemistry, National Centre of Competence in Research (NCCR) Chemical Biology, University of Geneva, Geneva, Switzerland

**Keywords:** automation, cell-penetrating peptides, cellular uptake, cytosolic delivery, cytotoxicity, high-content imaging, thiol-mediated uptake

## Abstract

Recent progress with chemistry tools to deliver into living cells has seen a shift of attention from counterion-mediated uptake of cell-penetrating peptides (CPPs) and their mimics, particularly the Schmuck cation, toward thiol-mediated uptake with cell-penetrating poly(disulfide)s (CPDs) and cyclic oligochalcogenides (COCs), here exemplified by asparagusic acid. A persistent challenge in this evolution is the simultaneous and quantitative detection of cytosolic delivery and cytotoxicity in a high-throughput format. Here, we show that the combination of the HaloTag-based chloroalkane penetration assay (CAPA) with automated high-content (HC) microscopy can satisfy this need. The automated imaging of thousands of cells per condition in multiwell plates allows us to obtain quantitative data on not only the fluorescence intensity but also on the localization in a very short time. Quantitative and statistically relevant results can be obtained from dose–response curves of the targeted delivery to selected cells and the cytotoxicity in the same experiment, even with poorly optimized cellular systems.

## Introduction

**Figure 1:**
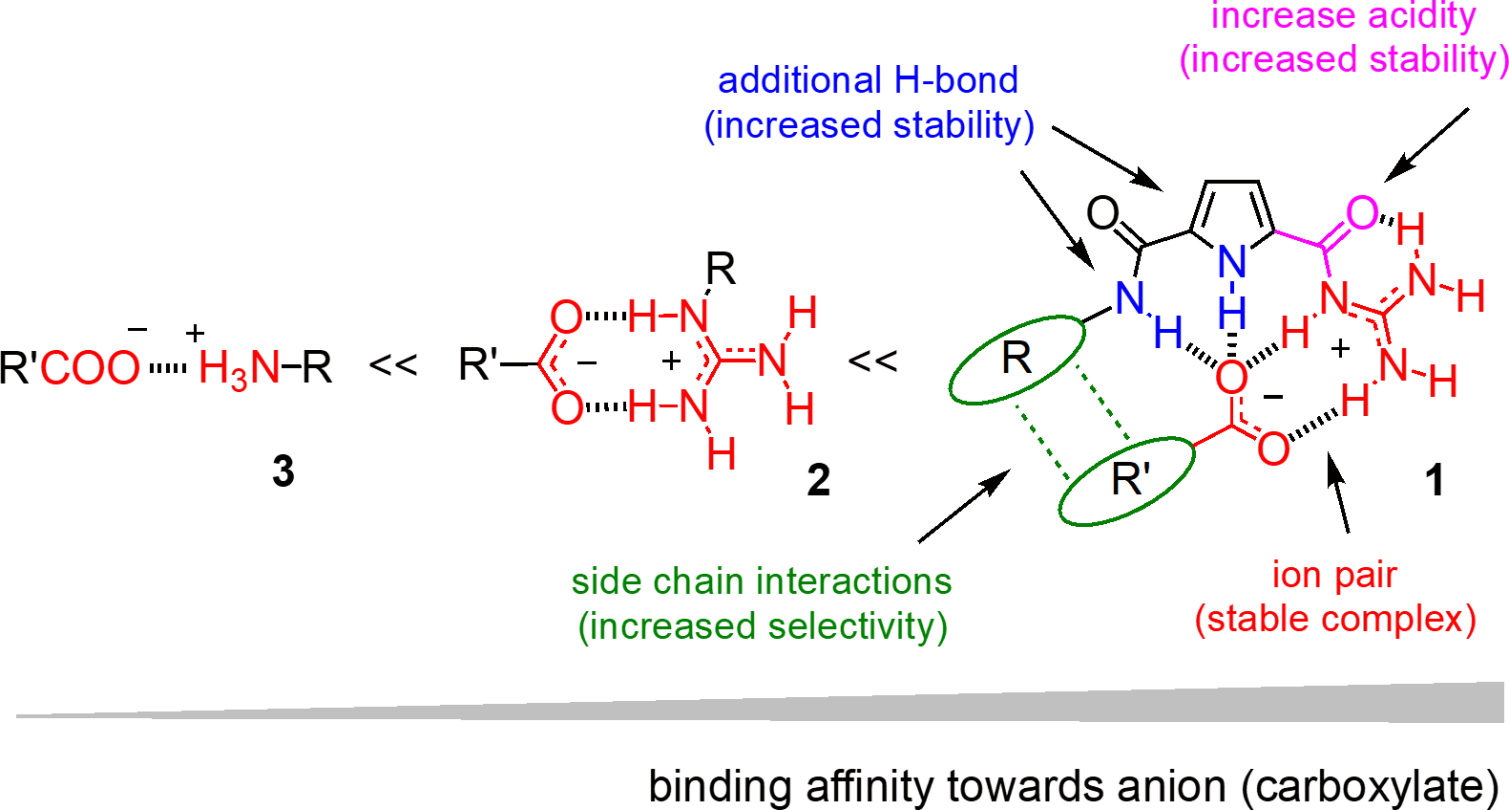
Schematic representation of binding models between organic cations (simple ammonium, guanidinium, Schmuck cation) and oxoanions.

The effective delivery of substrates of free choice into cells with minimal endosomal capture on the one hand and a minimal toxicity on the other remains one of the grand challenges in science [1–17]. This challenge is most pronounced with large substrates, such as proteins, oligonucleotides, or nanoparticles, due to the permeability barriers formed by the lipophilic core of the cell membrane [18–19]. In recent decades, the use of arginine-rich cell-penetrating peptides (CPPs) as carriers has emerged as an attractive approach to tackle this central challenge [1–3,11–14]. The noncovalent interaction between the guanidinium cations from CPPs and cell membrane-associated anions, such as phospholipids, proteoglycans, or sialic acids, is considered to enhance the cell surface accumulation of substrates, and thus fulfilling the first prerequisite of all internalization (Figure 1) [12,16–17,20–21]. However, this ion-pair interaction weakens significantly in polar solvents. The presence of competing anions in physiological solutions often disturbs the binding, and thus restricting the intracellular delivery to a certain extent.

In recent years, the development of artificial guanidinium systems with improved binding affinity and stability towards oxoanions has emerged as an important topic in this field [1,22–24]. In this context, Carsten Schmuck has created the 2-(guanidiniocarbonyl)pyrrole (GCP) cation **1** as a synthetic analogue of the guanidinium cations, somehow a “super-guanidinium” conceived to drive “arginine magic” [20–21] to the extreme (Figure 1) [23]. The power of the Schmuck cation to bind carboxylate and phosphate anions in competitive water has several origins [22]. Firstly, in comparison to simple guanidinium cations **2** (p*K*_a_ 12.5) and ammonium cations **3** (p*K*_a_ 10.5), the Schmuck cation has a lower p*K*_a_ value of 7 to 8 due to the increased acidity of acylguanidiniums, which favors the formation of stronger hydrogen-bonded ion pairs (Figure 1, magenta part). Secondly, the binding is further enhanced by the addition of hydrogen-bonding interactions between the amide NH moiety in position 5 of the pyrrole ring or the pyrrole NH group and the oxoanion (Figure 1, blue part). Thirdly, the rigid and planar conformation of the GCP moiety is beneficial to bind planar anions such as carboxylate (Figure 1, red part). Finally, the selectivity and specificity for different substrates can be achieved through the additional secondary interactions between the GCP side chain and the anionic substrates (Figure 1, green part).

**Figure 2:**
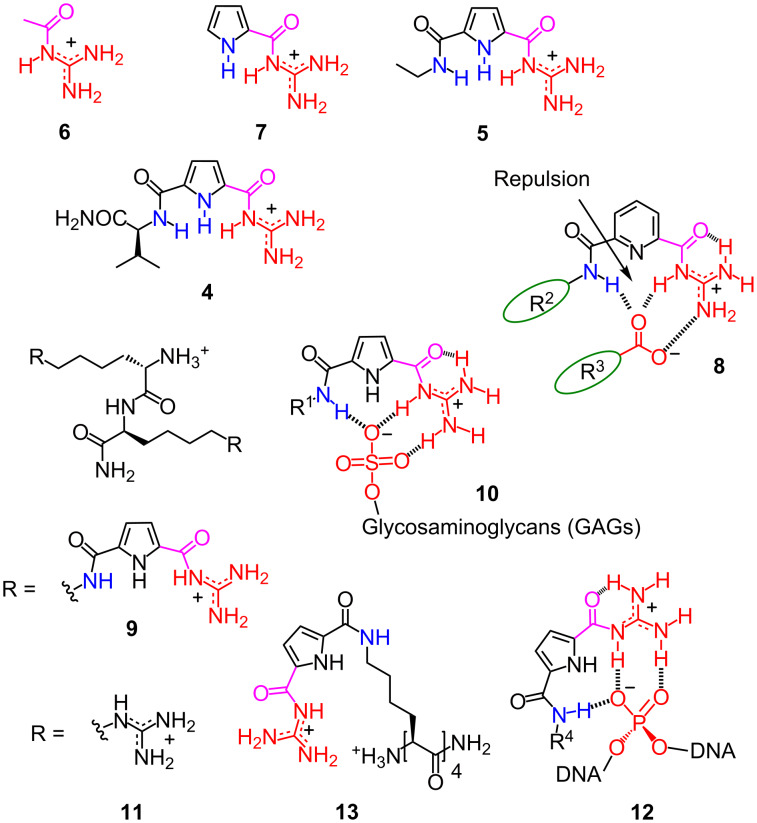
From Schmuck cations to cell-penetrating dipeptides, with schematic representation of the binding model between GCPs and GAGs on the cell surface, and with DNA.

The binding of the Schmuck cation with carboxylates in aqueous solvents was evaluated by a series of experimental studies, such as NMR, UV, CD, and fluorescence titrations [23,25]. The Schmuck cation indeed showed a much higher affinity towards carboxylates, with dissociation constants of *K*_D_ ≈ 1 mM (**4**: 620 µM; **5**: 1.3 mM) compared to simple acylguanidinium cations (**6**: *K*_D_ = 20 mM, Figure 2). The amide NH unit in position 5 of the pyrrole ring is crucial for this binding affinity, which even exceeds the effect of the pyrrole NH moiety (**4** and **5** vs **7**: *K*_D_ = 7.7 mM). In addition, the size and electronic structure of the pyrrole core are also important for this ion-pair interaction. For example, the replacement of the pyrrole core to pyridine in **8** or to furan results in a much weaker binding because of the repulsion force between the lone pair on the heteroatom and the oxoanion.

The superior oxoanion binding of the Schmuck cation makes GCP-based peptides attractive candidates for intracellular delivery. Already a small dipeptide **9** with two Schmuck amino acids shows a strong binding (*K*_D_ = 100 nM) and clustering ability towards heparin due to the strong noncovalent interaction between GCP and sulfate anions, such as glycosaminoglycans (GAGs) in heparin [26]. This binding model **10** can be applied to cell-surface GAGs to enhance the cellular uptake efficiency. The peptide **9**, with rhodamine B attached, successfully enters into the living cells while the control peptide **11** with a simple guanidinium group shows a negligible uptake efficiency. In addition, the efficient delivery of a model protein (avidin, around 67 kDa) into cells through biotin–avidin technology could be achieved in the presence of this strikingly small peptide. However, the uptake of peptide **9**-labeled avidin was dramatically reduced into cells that express less GAGs on the cell surfaces. These results further support the importance of GAG binding to the uptake of Schmuck cations.

Arginine-rich CPPs are of general interest in gene delivery. However, a long linear CPP sequence with at least eight to nine arginine residues is necessary. In comparison to arginine-rich CPPs, Schmuck peptides form more stable complexes **12** with the phosphodiesters in the DNA backbone, and thus making it possible to transfect cells with shorter peptides. In 2015, the Schmuck group reported the first example of a small peptide with only four amino acids for gene transfection [27]. The binding affinity of **13** to DNA far exceeds the related tetrapeptide analogues with arginine or lysine residues. As a result, the gene transfection efficiency of **13** is better than that of polyethylenimine (PEI) with a large number of charges, which is one of the current standards in gene transfection. The uptake takes place through an endosomal pathway. The low p*K*_a_ value of the four GCP moieties could result in an improved buffering capacity, which could facilitate endosomal escape by the proton-sponge effect [28]. Significant inhibition of DNA transfection by bafilomycin (a macrolide antibiotic that can block the endosomal acidification process) was observed, which further supports the endosomal uptake mechanism. Except for this small peptide, GCP was also integrated into larger peptides, including branched [29], three-armed [30–31], dendritic [32], and self-assembled oligomers [33–34] for gene delivery and transfection based on the endosomal uptake and release.

**Figure 3:**
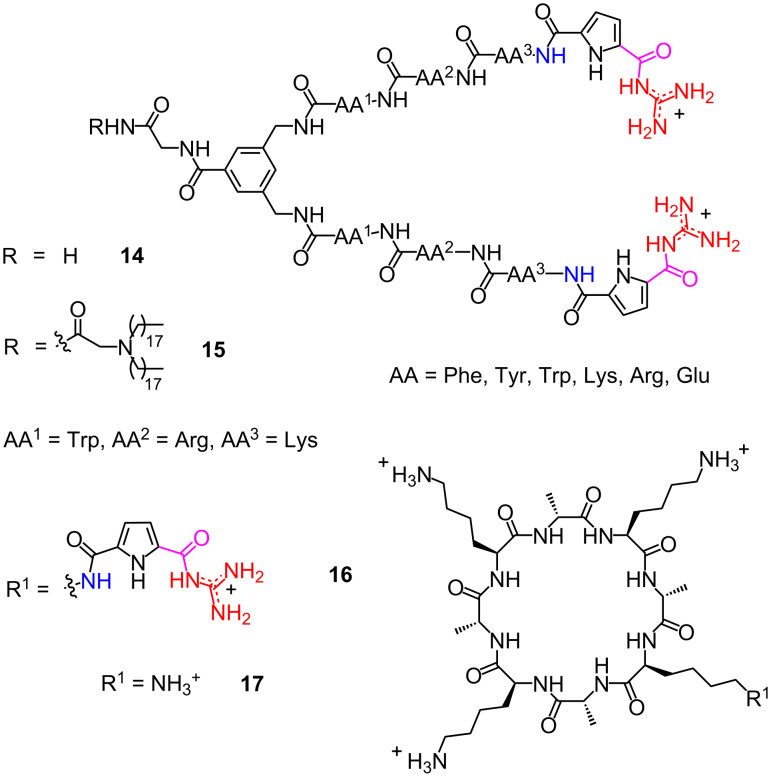
Peptide tweezers and cyclic peptides with Schmuck cations for gene transfection.

However, a better binding affinity to DNA does not necessarily mean a higher DNA transfection efficiency. As an example, two GCP-modified peptide tweezers **14** with nanomolar dissociation constants, identified by the high-throughput screening of a combinatorial library of 259 molecular tweezers through an ethidium bromide (EB) displacement assay, show a negligible DNA transfection efficiency (Figure 3). However, the derivative **15**, with two lipophilic hydrocarbon chains, results in a remarkable DNA delivery and transfection [35]. These two hydrocarbon chains attached to the tweezers are used to facilitate endosomal escape. These results indicate that the balance between the number of GCP, the binding affinity, and the buffering capacity of Schmuck peptides plays a key role in the gene transfection process.

In 2016, the Schmuck group developed the first cyclic peptides that can be used as gene transfection vectors [36]. Unlike linear Schmuck peptides, the cyclic peptide **16** can self-assemble into nanofibers due to the binding between the GCP moiety and the backbone of an adjacent peptide. This binding interaction could stabilize the stacking of peptides by offsetting a charge repulsion of the extra lysine residues, and thus allowing the formation of stable cationic nanofibers. These nanoaggregates, assembled from the monomer **16**, are efficient gene transfection vectors. However, the control peptide **17**, which cannot self-assemble into nanotubes, shows negative transfection results. In contrast to the linear Schmuck peptides, the inactivity of a bafilomycin treatment in gene transfection processes indicates the nonendocytic cellular uptake pathway.

**Figure 4:**
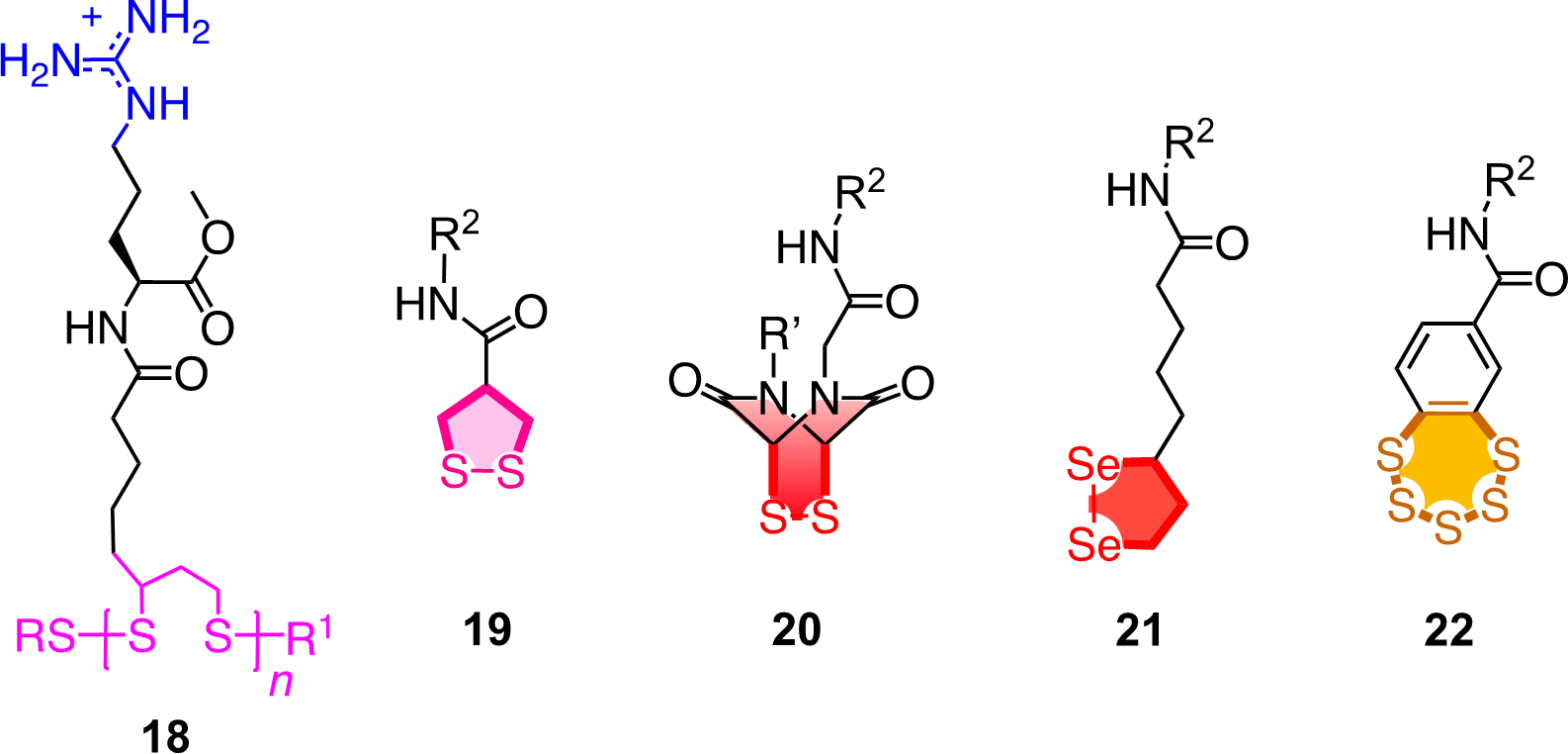
Evolution from CPPs to CPDs and COCs.

**Figure 5:**
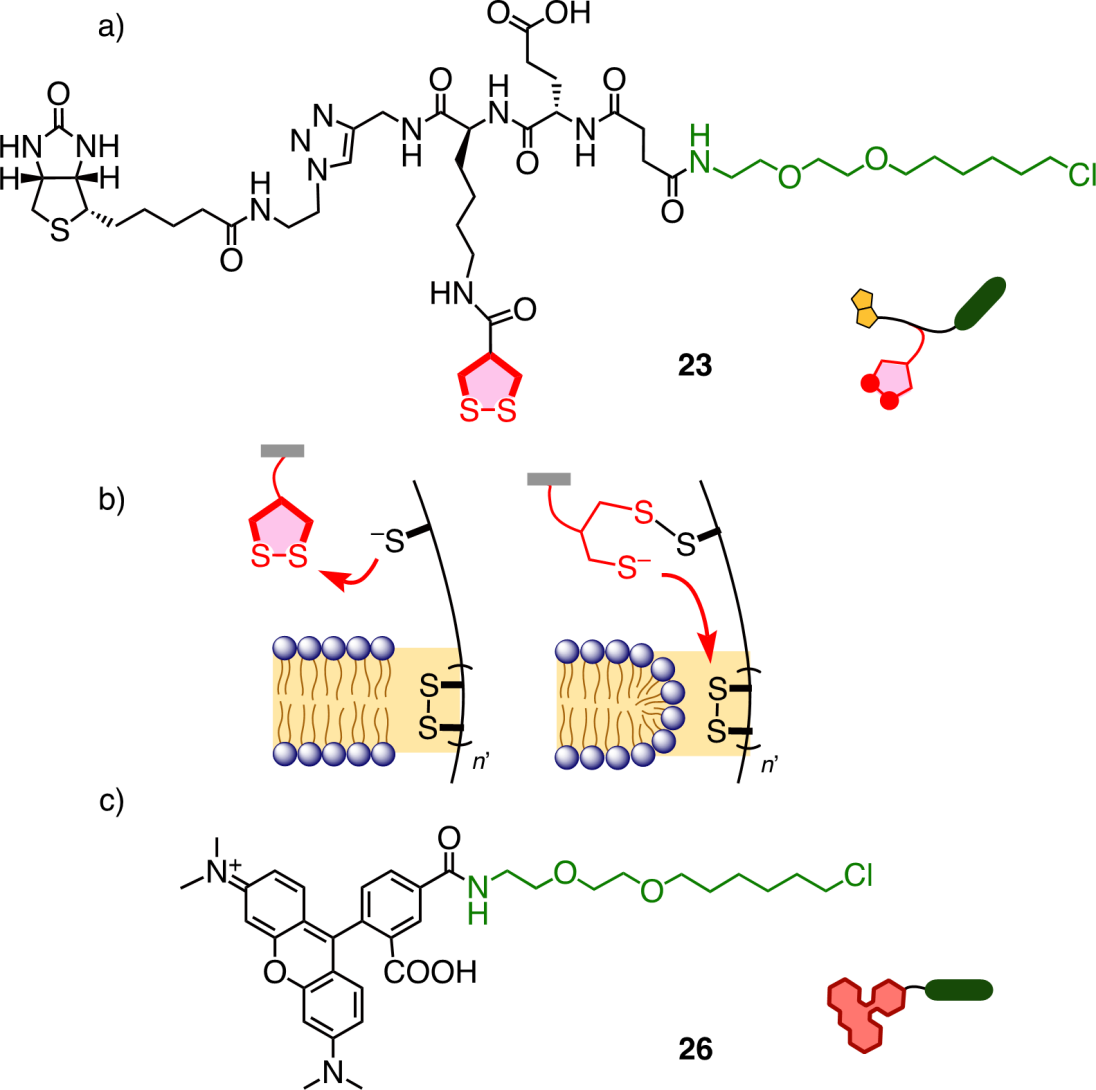
Structure of a) the trifunctional transporter **23** and c) the HaloTag reporter **26**. b) Schematic mechanism of the thiol-mediated uptake of COCs with dynamic covalent disulfide exchange with exofacial thiols (left), preceding walking along the disulfide tracks, and micellar pores (right).

Among the central challenges with CPPs in general are the cytotoxicity and the endosomal capture, particularly with an increasing number of charges and substrate size [18]. To address both problems, the peptide backbone has been replaced by poly(disulfide)s [18,37]. The resulting cell-penetrating poly(disulfide)s (CPDs) **18** are at least as efficient as CPPs but less toxic because they are degraded by reductive depolymerization as soon as they reach the cytosol, and their endosomal capture is minimal because they enter cells by thiol-mediated uptake (Figure 4) [38–48]. This mechanism operates by dynamic covalent disulfide exchange on the cell surface and on the way into the cell (Figure 5b) [49–50]. The cyclic oligochalcogenides (COCs) **19**–**22** were introduced to maximize the speed and selectivity of this dynamic covalent exchange chemistry for an efficient cytosolic delivery (Figure 4) [51]. Moving from the original emphasis on the disulfide ring tension into an increasing unorthodox COC chemistry covering ETPs [52], diselenolanes [53], and benzopolysulfanes [54], the activity gradually increased.

The Achilles heel of much research on new transporters for cellular uptake, from CPPs and Schmuck cations to CPDs and COCs, is the quantitative detection of the delivery in the intact functional form to the cytosol, firmly excluding possible false positives from endosomal capture on the one hand and cytotoxicity on the other [5,55]. Recently, the CAPA has been introduced to quantify cytosolic delivery [55]. The combination with image-based high-content (HC) screening [56–58] has been suggested to further improve the standards set by the CAPA [59]. Standard high-throughput (HT) screening has been used regularly to facilitate studies on cellular uptake [60]. In standard assays, a single macroscopic parameter, usually the fluorescence intensity, is automatically recorded for microtiter plates with hundreds to thousands of wells. This standard HT screening provides access to quantitatively reliable curves for the dependence on the concentration, incubation time, activators, inhibitors, and so on within a reasonable time. High-content screening (HCS) combines this automated HT format with image-based information.

To summarize briefly what has been outlined previously in more detail [56], HCS requires automated high-speed microscopy, including robotic liquid handlers and plate washers, automated data analysis tools, and large data storage systems for the terabits of data produced per day. The high number of images generated by the automated microscope cannot be analysed manually, and therefore need to go through a completely automated analysis pipeline. For this purpose, dedicated software is used to first create specific masks that detect the objects of interest in the image (e.g., cells, cellular organelles, etc.) This can be achieved by using several steps of image curation and modification (e.g., deconvolution) and using several analysis modules adapted to the shape, size, and intensity of the object of interest. Once all masks are properly detected and associated to the master object (the cell), a set of meaningful data can be extracted from each object in the different fluorescent channels. The data set generated for each cell can later be used for a deep phenotypic analysis, using an advanced unsupervised statistical analysis method (e.g., principal component analysis, diverse clustering methods, etc.), or for directly plotting specific parameters of interest. This fully automated workflow allows then to extract deep complex information from thousands of cells extremely rapidly and in a very consistent manner, allowing a very efficient comparison of the cell treatment conditions and sequential experiments. For cellular uptake, this promises the recording of statistically relevant dose–response curves for a targeted delivery, together with other characteristics of interest. This can include the incubation time or a cytotoxicity that is precisely defined on the changes in the appearance of interest. In the following, these expectations are evaluated explicitly, with a particular emphasis on quantifying the eventual contributions from off-target fluorescence as well as extracting quantitative information on the cytosolic delivery and cell viability in one and the same automated HC screen.

## Results and Discussion

The new, concise, at least trifunctional COC transporter **23** was designed and synthesized to explore the usefulness of a HC screening to study the cellular uptake (Figure 5a). Asparagusic acid was chosen as the arguably best explored COC for the thiol-mediated uptake, beginning with a dynamic covalent disulfide exchange with exofacial thiols, followed by either endocytosis or the direct crossing of membranes into cytosols through successive thiolate–disulfide exchange reactions or micellar pores (Figure 5b) [61]. Anionic glutamate was added to minimize the passive diffusion across the membrane and to maximize the solubility in water. Biotin was used to interface the transporter and streptavidin to probe the COC-mediated protein delivery. A chloroalkane, finally, was needed for the HC CAPA.

**Figure 6:**
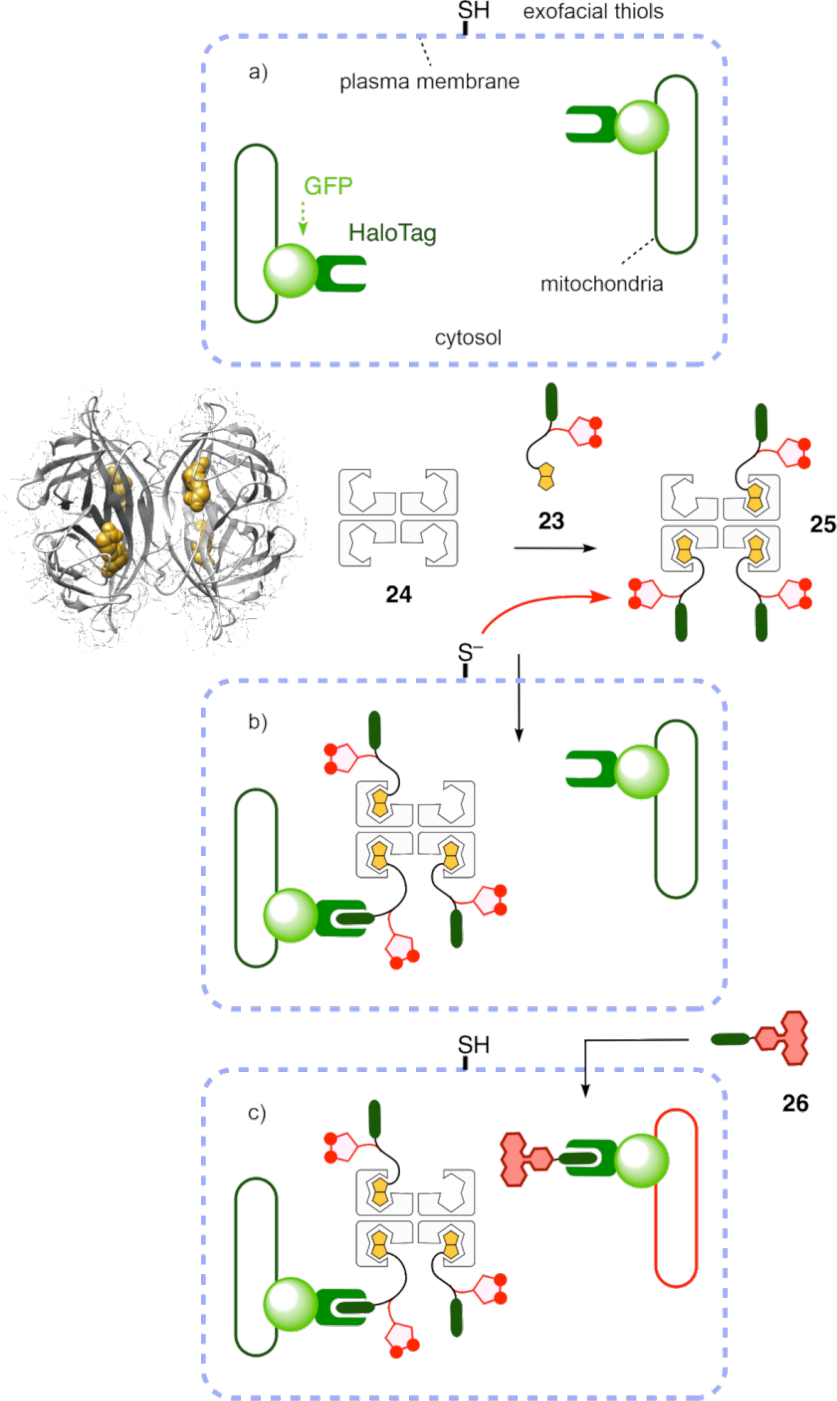
CAPA assay for the complex **25**, composed of three transporters **23** bound to one streptavidin **24** (with the structure of the homotetramer loaded with four biotins). Cytosolic delivery into HMG cells (a) has to precede the reaction of **25** with HaloTags on mitochondria (b). Afterwards, the unreacted HaloTags are labeled with the reporter **26** for quantification (c).

The trifunctional transporter **23** was first complexed with streptavidin **24** (Figure 6). The resulting complex **25** was then incubated with HGM cells. These are stable cell lines, expressing the self-labeling HaloTag protein and GFP in the outer mitochondrial membrane (Figure 6a) [55]. If the complex **25** indeed reaches the cytosol, the chloroalkane will react with carboxylate in the active site to produce an ester, and thus covalently attach the transporter to the fusion protein (Figure 6b). The subsequently added reporter **26** passively diffuses into the cells and labels all free HaloTags. The fluorescence signal from the HaloTag reporter **26** is then employed to generate dose–response curves and to calculate the CP_50_ value of the transporters, described as the half-maximal cell penetration.

**Figure 7:**
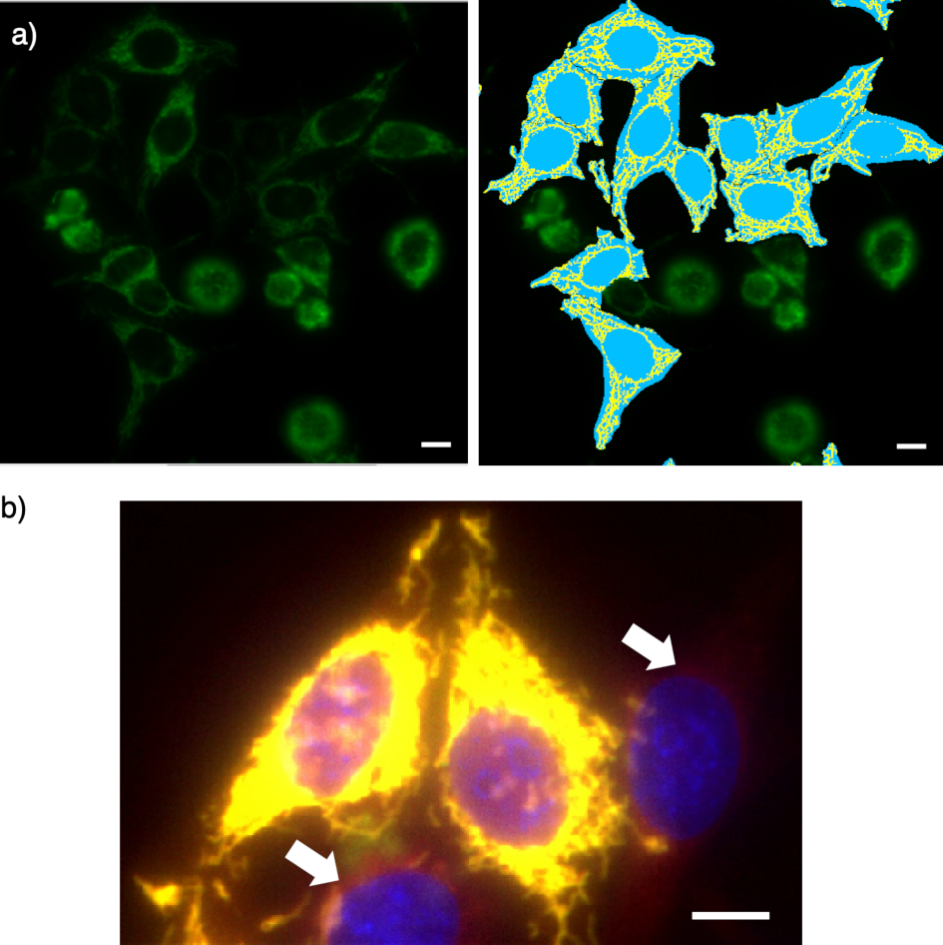
Examples from the automated HC imaging of stable HGM cells with HaloTag–GFP on mitochondria, labeled with **26** and Hoechst dye. a) GFP channel (left) and cell selection (right), showing the cell body mask (light blue areas) and the mitochondrial mask (yellow areas). b) Overlay image of the GFP (green), **26** (red), and the Hoechst dye (blue) channels, showing off-target fluorescence from **26** (white arrows). Scale bar: 10 µm.

In a CAPA, the final fluorescence response is usually recorded by flow cytometry [55,62]. In a HC CAPA, flow cytometry is replaced by HC automated microscopy, using multiwell plates and registering data on the fluorescence intensity and fluorescence localization in thousands of cells per condition, at HT [56–59]. To properly assess its potential, automated HC imaging was optimized first. The fluorescence of the Hoechst dye and the GFP of the fusion protein were used to segment whole cells and mitochondria, respectively (blue and yellow areas, Figure 7a). The structural characteristics of the cells were extracted from both masks.

Possible damaged cells with early signs of apoptosis or abnormal mitochondrial networks were excluded (Figure 7a, right). With these criteria, on average, 15% of the cells were discarded. This possibility to simultaneously quantify the cytotoxicity and the cytosolic delivery in the same HT experiment is one of the key advantages of the HC CAPA (vide infra).

**Figure 8:**
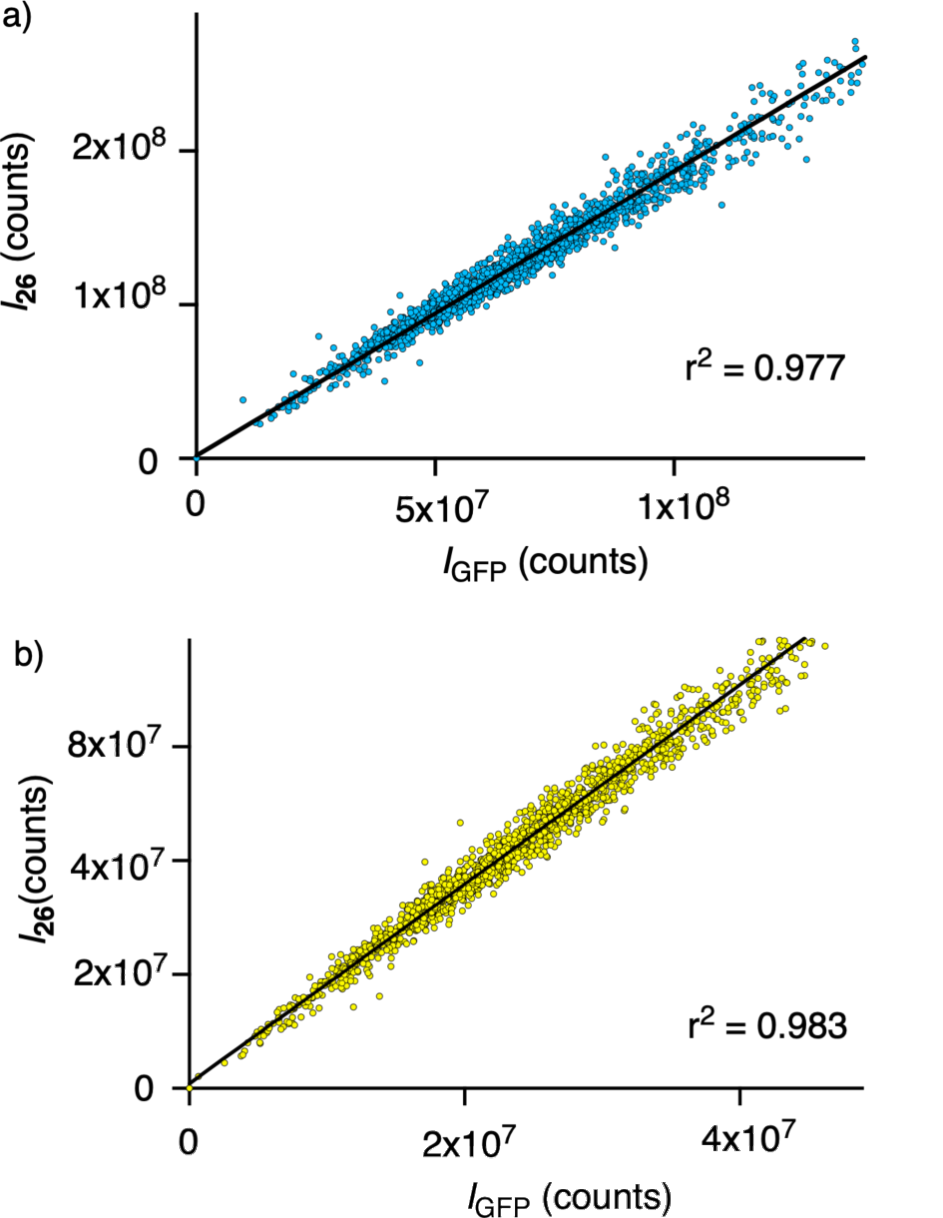
Evaluation of the automated HC imaging of stable HGM cells with HaloTag–GFP on mitochondria, labeled with **26**, showing the fluorescence intensity of GFP versus **26** in a) selected cells inside the cell body mask (light blue and yellow areas in Figure 7a) and in b) selected regions with a high GFP signal, considered as the mitochondrial network (yellow areas only in Figure 7a).

After the addition of the reporter **26**, a large-scale analysis was carried out, correlating its fluorescence intensity with that of the GFP, proportional to the concentration of the HaloTag, cell by cell. A linear regression of the correlations using the whole-cell body as a mask gave a very good r^2^ of 0.977 (Figure 8a). The same linear regression of correlations, masking exclusively the mitochondria region, gave a small but significant increase to r^2^ = 0.983 (Figure 8b). Although optimized toward perfection with stable HGM cells, this increase demonstrated that the HC CAPA adds a precision that is overlooked with flow cytometry. The off-target staining of **26** in regions outside mitochondria accounted for this source of error in the CAPA, which is corrected in the HC CAPA (Figure 7b, arrows).

**Figure 9:**
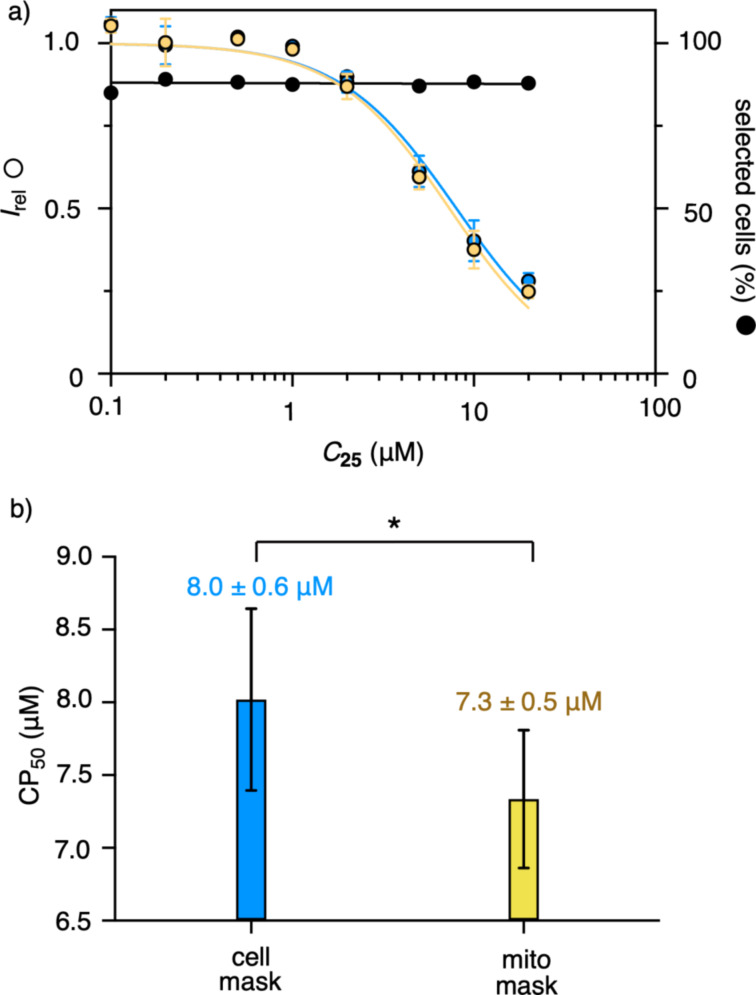
a) Automated HC imaging of the cellular uptake of **25**, covering the concentration dependence for the HC CAPA with the cell body mask (blue), the HC CAPA with the mitochondrial mask (yellow), and the toxicity defined as the percentage of damage-free cells selected for the HC CAPA (black), all recorded in one automated experiment. (b) The CP_50_ value of **25** with standard errors using the cell body mask (blue) and the mitochondrial mask (yellow). The statistical significance was determined using the one-tailed paired Student’s t-test: *p < 0.05.

The optimized HC CAPA system was then explored with the complex **25**, recording the cytosolic delivery and the cytotoxicity quantitatively in one HT experiment. A nearly constant number of selected cells based on the above criteria, with an increasing concentration, revealed that complex **25** is not toxic up to 20 µM (Figure 9a, black). The effective concentration of **25** obtained with the mitochondria mask was CP_50_ = 7.3 ± 0.5 μM (Figure 9a and Figure 9b, yellow). With the whole-cell mask, a CP_50_ value of 8.0 ± 0.6 μM was obtained (Figure 9a and Figure 9b, blue). According to *p* < 0.05, the underestimate made with the whole-cell mask was significant (Figure 9b). Namely, the cytosolic delivery of **25** measured with the mitochondria mask was i) 10% better and ii) also 10% more accurate. These small but significant differences demonstrated that the HC CAPA improves even on a cytometric CAPA, although the optimization of this assay with stable HGM cells is near perfection.

The CP_50_ value of 7.3 ± 0.5 µM obtained for **25** outperformed the CP_50_ value of 14.9 ± 0.5 μM of the previously reported cell-penetrating streptavidin, in which four asparagusic acids are covalently bound to the protein through irreversible triazole linkages [59]. The CP_50_ value of 7.3 ± 0.5 µM of **25** was not far above the CP_50_ value of 3.1 ± 0.5 µM of HIV Tat, the original CPP [55]. As the uptake efficiency generally decreases with the size [18], this similarity is particularly impressive, considering that the COC carrying a 52 kDa protein is compared to a small undecapeptide.

The compatibility of the assay with other fusion proteins and more adverse conditions, such as transient transfection, has never been assessed for the standard cytometric CAPA, but a low precision and reproducibility were anticipated [62]. The high accuracy and selectivity promised that our image-based HC CAPA could expand the scope of the CAPA beyond HGM cells. To elaborate on this attractive perspective, HeLa cells were transiently transfected with a plasmid expressing a fusion protein of HaloTag–GFP–Golgi localization sequence [63]. The HaloTags installed in the Golgi were then labeled with the reporter **26**.

**Figure 10:**
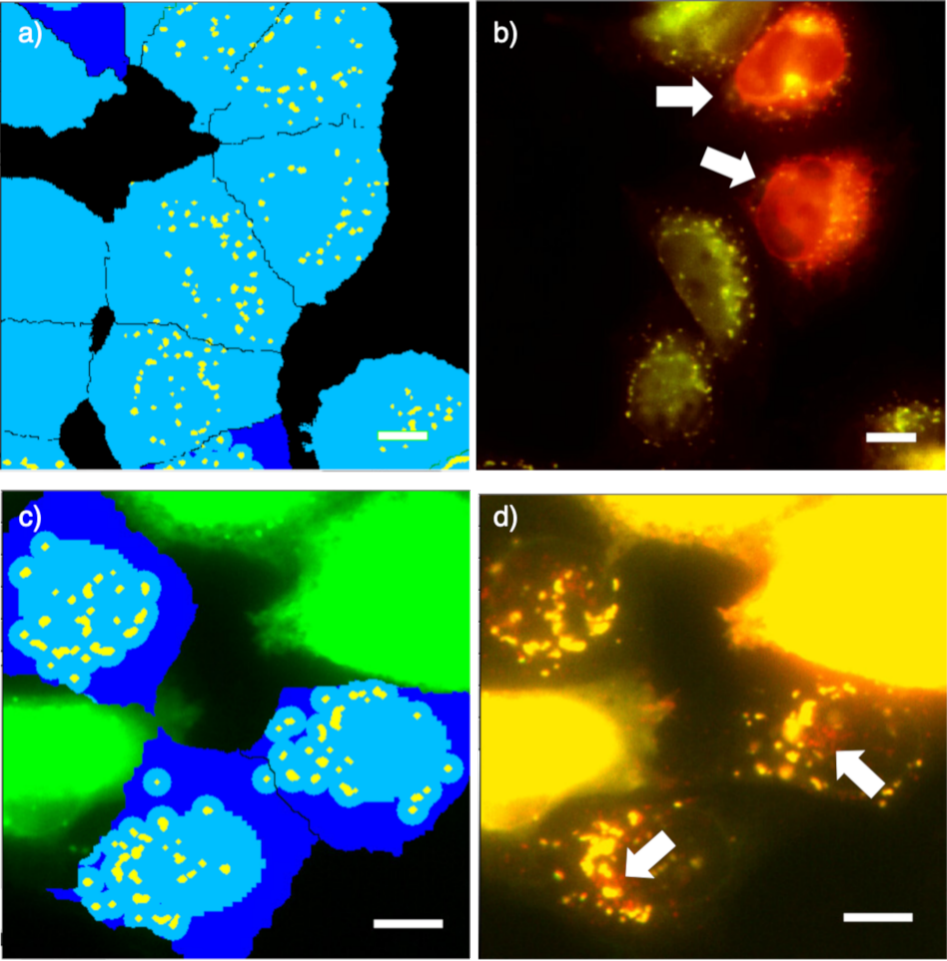
Examples of automated HC imaging of transiently transfected HeLa cells with HaloTag–GFP on Golgi, labeled with **26**. a) GFP channel showing the applied cell-body mask (light blue), Golgi mask (yellow), and the mask of the cell regions with low or no GFP signal (dark blue) in all cells. b) Same image as in a) with an overlay (yellow) of the GFP (green) and **26** (red) channels, showing off-target fluorescence from **26** in some cells (white arrows). c) GFP channel after the selection of the cells with an adequate level of expression of the fusion protein. The applied masks for the cell body (light blue), the Golgi (yellow), and the cell regions with low or no GFP signal (dark blue) are shown only in the selected cells. Over-transfected cells (without dark blue regions) are not masked and excluded from the analysis. A very intense GFP signal (green) can be seen in the excluded cells. d) The same image as in c) with an overlay (yellow) of the GFP (green) and **26** (red) channels, showing off-target punctate fluorescence from **26** in some specific locations inside the selected cells (white arrows). Scale bar: 10 µm.

**Figure 11:**
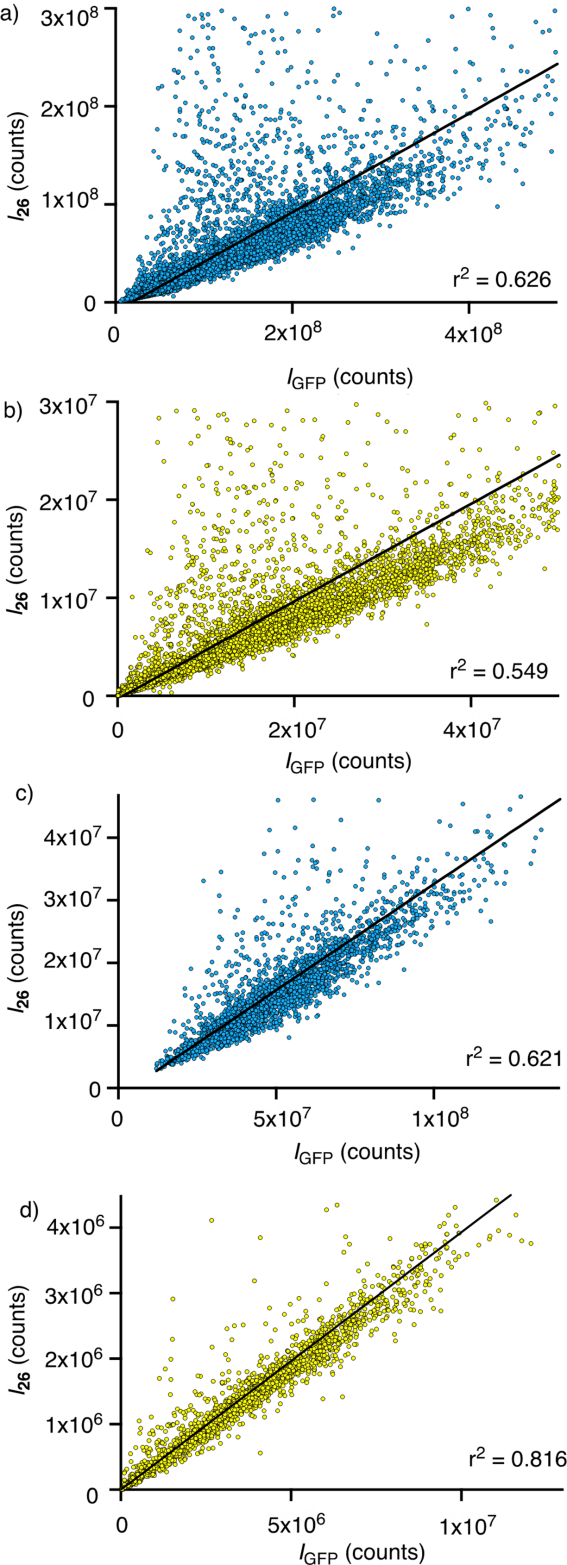
Evaluation of the automated HC imaging of the transiently transfected HeLa cells with HaloTag–GFP on Golgi, labeled with **26**, showing the fluorescence intensity of GFP versus **26** in a) all cells inside the cell body mask (yellow and blue areas in Figure 10a), b) all cells inside the punctate regions with a high GFP signal, considered as the Golgi apparatus (yellow areas in Figure 10a), c) selected cells inside the cell body mask (light and dark blue areas in Figure 10c), and d) punctate regions with a high GFP signal in selected cells, considered as the Golgi apparatus (yellow areas in Figure 10c).

Under these more challenging conditions, obvious problems arose from the toxicity of the transfecting agent, over- and underexpression of the fusion protein, and undesired localization out of the selected organelle. To explore how automated HC imaging can handle these problems, a whole-cell body mask was applied first (Figure 10a, cyan, including yellow). With this mask, the fluorescence of GFP and **26** correlated with r^2^ = 0.626 (Figure 11a). This very poor correlation for the transient transfection of the HaloTags on the Golgi contrasted sharply with an r^2^ value of 0.983 obtained with the stable HGM cells with the HaloTags expressed on the mitochondria. The difference between the two plots in Figure 11a and Figure 8a was striking even for the naked eye. A comparison of the respective images confirmed a massive off-target emission of **26** in the former, with r^2^ = 0.626 (Figure 10b, red, arrows) and little off-target emission in the latter, with r^2^ = 0.983 (Figure 7b, red, arrows).

Masking of the regions with the intense GFP signal from the Golgi apparatus without an initial cell selection was tested next (Figure 10a, yellow only). With this Golgi mask without cell selection, the correlation of the fluorescence of the GFP and **26**, with an r^2^ value of 0.549, was even worse than the r^2^ value of 0.626 for the whole-cell mask (Figure 11b).

The initial cell selection aimed to narrow the polydispersity of expression and to exclude the overtransfected and damaged cells. For this purpose, cells with GFP fluorescence in all cell bodies were removed, and only those cells with nonfluorescent regions were kept (dark blue regions in Figure 10c). This selection removed about 80% of all cells, and only the top 20% were kept for further analysis. However, applying a cell body mask to this remaining 20%, the fitting of r^2^ = 0.621 of the GFP and the **26** emission did not improve either (Figure 10c, cyan, including yellow and Figure 11c). The inspection of the images confirmed that even the selected cells showed a significant off-target emission of **26** (Figure 10d, red, arrows).

The masking of the Golgi apparatus, based on the GFP emission in the selected cells, was applied next to remove this off-target staining from **26** (Figure 11c, yellow only). The application of both the cell selection and the Golgi mask to the analysis finally improved the correlation of the GFP and the **26** emission to r^2^ = 0.816 (Figure 11d). This value was slowly approaching the precision realized without further effort using the stable cell line HGM (Figure 8b). While the error from the whole-cell analyses with HGM cells is not catastrophic (r^2^ = 0.977 vs r^2^ = 0.983), these results show that automated high-content imaging is absolutely necessary with less optimized systems (r^2^ = 0.626 vs r^2^ = 0.816).

The difference between the optimized HGM cells and the unoptimized transfected cells is again best appreciated with the naked eye: Whereas the improvement of the automated selection from Figure 8a to Figure 8b is not visible on the first view, the improvement from Figure 11a to Figure 11d is massive, and Figure 11d starts to resemble more the quasiideal Figure 8b. The automated removal of 80% of all cells to obtain Figure 11d (compared to 15% to produce Figure 8b) is not problematic because HC microscopy registers, screens, and evaluates the data after the optimization of the ideal conditions for the analysis [56–59].

## Conclusion

The specific objective of this study was to assess the power and uniqueness of HC imaging for cellular uptake. For this, the HC CAPA, which combines automated microscopy and precise localized quantification, has been first improved using a cell selection process, aiming at removing damaged or abnormal cells (around 15%). The correlation of the GFP fluorescence (proportional to the HaloTag expression) and the fluorescence of the CAPA assay reporter **26** is identified as a practical method to assess the accuracy of the mask analysis. After a linear regression, a slight increase in the goodness of fit from r^2^ = 0.977 to 0.983 quantified the increase in accuracy achieved moving from a cell-body mask to a mitochondrial mask. This difference revealed that even with fully optimized stable HGM cell lines, whole-cell analyses, such as flow cytometry, of the CAPA contain a small but nonnegligible error that can be removed with the HC CAPA.

To quantify the impact of the HC CAPA on the detection of the cytosolic delivery, the newly introduced peptide-based COC **23** was evaluated for the transport of a model protein with the new analytical improvements. With a CP_50_ value of 7.3 ± 0.5 μM with the mitochondrial mask, complex **25** showed an excellent transport efficiency. A slightly larger CP_50_ value of 8.0 ± 0.6 μM obtained with the whole-cell mask confirmed that the error from the whole-cell analyses, such as flow cytometry, in the CAPA is small but not negligible (i.e., 10% for the activity, 10% for the accuracy). The CP_50_ value obtained for the COC–protein complex was in the range of the protein-free HIV Tat, and thus confirming the excellent activity of the COCs. Most importantly, with our refined HC CAPA, quantitative dose–response curves for the cytotoxicity could be obtained with the same HT screening experiment, quantifying the cytosolic delivery, and the cytotoxicity can be defined according to the cellular defects of interest. The COC–protein complex **25** was nontoxic up to at least 20 µM.

Finally, the HC CAPA is not limited to optimized stable cell lines to generate precise and reproducible data. The compatibility with transient transfection is exemplified with HeLa cells with a GFP–HaloTag construct in the Golgi apparatus, after the application of the cell selection process and a Golgi mask to the analysis. As a result, over- and underexpressing cells, around 80% of the total cells, could be efficiently removed from the analysis, and the localized fluorescence in the selected cells could be quantified with the Golgi mask. The goodness-of-fit after a linear regression (r^2^ = 0.816) of the correlation between the GFP fluorescence and the reporter **26** in transiently transfected cells was increasingly close to the one obtained with the HGM stable cell line (r^2^ = 0.983).

These results imply that automated HC microscopy in general and the HC CAPA in particular can be used as a universal tool to quantify the cellular uptake, including transiently transfected cells and other less robust systems. Quantitative, statistically relevant data on the targeted delivery and a precisely defined cytotoxicity are obtained in the same HT experiment. We hope that automated HC microscopy will be useful for the community to routinely assess synthetic transport systems, from the Schmuck cation to the latest CPPs, CPDs, and COCs.

## Supporting Information

File 1 Experimental details.
