# Supplementary material for: Automated high-content imaging for cellular uptake, from the Schmuck cation to the latest cyclic oligochalcogenides
Source: Beilstein J Org Chem. 2020 Aug 14;16:2007–16. doi: 10.3762/bjoc.16.167 (PMC7431755; doi:10.3762/bjoc.16.167)
Supplement: File 1 — Experimental details. [file Beilstein_J_Org_Chem-16-2007-s001.pdf]

BEILSTEIN JOURNAL OF ORGANIC CHEMISTRY

Supporting Information

for

Automated high-content imaging for cellular uptake, from the Schmuck cation to the latest cyclic oligochalcogenides

Rémi Martinent, Javier López-Andarias, Dimitri Moreau, Yangyang Cheng, Naomi Sakai and Stefan Matile

Beilstein J. Org. Chem. **2020,** *16,* 2007–2016. doi:10.3762/bjoc.16.167

Experimental details

License and Terms: This is a supporting information file under the terms of the Creative Commons Attribution License (http://creativecommons.org/licenses/by/4.0). Please note that the reuse, redistribution and reproduction in particular requires that the authors and source are credited.
The license is subject to the *Beilstein Journal of Organic Chemistry* terms and conditions: (https://www.beilstein-journals.org/bjoc)

Table of Contents

1	Materials and methods	S2
2	Synthesis	S3
3	HGM cell line and transient transfection protocol	S5
4	HC CAPA optimization	S6
4.1	General HC CAPA protocol	S6
4.2	Optimization of the HC CAPA data analysis in HGM cells	S7
4.3	HC CAPA for transient transfection	S10
4.4	Data analysis of the HC CAPA for transient transfection	S10
4.5	HC CAPA results for the uptake quantification	S13
5	References	S14
6	NMR spectra	S15
7.	LC–MS spectra	S16

1 Materials and methods

As in [S1]. Briefly, reagents for the synthesis were purchased from Fluka, Sigma-Aldrich, TCI and Across. Wild-type streptavidin was a generous gift from Prof. Thomas R. Ward (University of Basel). Phosphate-buffered saline (PBS, pH = 7.4), DMEM (GlutaMAX, 4.5 g/L D-glucose, with phenol red) medium, FluoroBrite DMEM (high D-glucose) medium, Leibovitz's L-15 medium, Opti-MEM reduced serum medium with GlutaMAX supplement, penicillin-streptomycin, fetal bovine serum, TrypLE Express Enzyme, and Lipofectamine 2000 were obtained from Thermo Fisher Scientific. μ-Plates 96-well were obtained from Ibidi. Fluorescence cellular imaging was performed using an IXM-C automated microscope from ImageXpress equipped with a Lumencor Aura III with 5 independently selectable solid-state light sources, bandpass filters and 5 objectives (4× to 60×). Sample preparation and washing on 96-well plates was performed using a Plate washer Biotek EL406®. IR spectra were recorded on a Perkin Elmer Spectrum One FT-IR spectrometer (ATR, Golden Gate) and are reported as wavenumbers ν in cm^{-1} with band intensities indicated as s (strong), m (medium), w (weak). ^{1}H and ^{13}C spectra were recorded on a Bruker 500 MHz spectrometer and are reported as chemical shifts (δ) in ppm relative to TMS (δ = 0). Spin multiplicities are reported as a singlet (s), doublet (d), triplet (t) and quartet (q) with coupling constants (*J*) given in Hz, or multiplet (m). Broad peaks are marked as br. ^{1}H and ^{13}C resonances were assigned with the aid of additional information from 1D and 2D NMR spectra (H,H-COSY, DEPT-135, HSQC and HMBC). LC–MS were recorded using a Thermo Scientific Accela HPLC equipped with a Thermo C18 (5 cm × 2.1 mm, 1.9 μm particles) Hypersil gold column coupled with an LCQ Fleet three-dimensional ion trap mass spectrometer (ESI, Thermo Scientific) with a linear elution gradient from 95% H_2O/5% CH_3CN + 0.1% TFA to 10% H_2O/90% CH_3CN + 0.1% TFA in 4.0 min at a flow rate of 0.75 mL/min. HRESIMS for the characterization of new

compounds was performed on a Xevo G2-S Tof (Waters) and are reported as the mass-per-charge ratio *m*/*z* calculated and observed.

Abbreviations. CAPA: chloroalkane penetration assay; CP_{50}: half maximal cell penetration concentration; DMEM: Dulbecco's modified eagle medium; DMSO: dimethyl sulfoxide; GFP: green fluorescent protein; HC: high-content; LP: laser power; PBS: phosphate-buffered saline; rt: room temperature; WT: wild type.

2 Synthesis

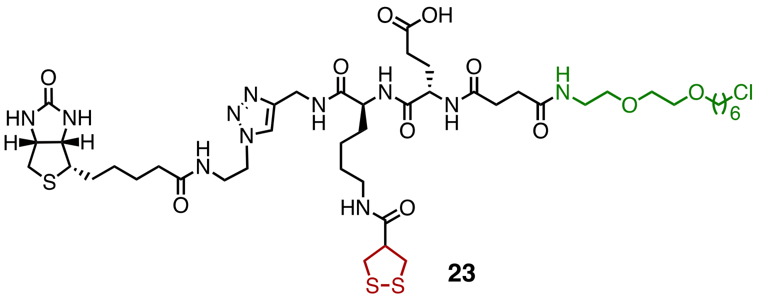

23

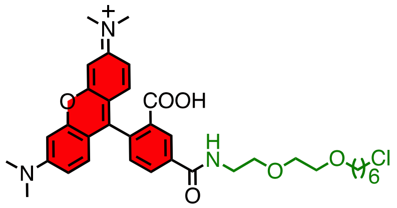

26

Figure S1: Structures of **23** and **26**.

Compound 23. The synthesis of this compound will be described elsewhere [S2]. IR (neat): 3282 (br), 2935 (m), 1635 (s), 1549 (s), 1432 (m), 1265 (m), 1094 (m), 568 (s), 536 (s); ^{1}H NMR (500 MHz, DMSO-d_6): 12.12 (br s, 1H), 8.18 (d, $^{3}J_{H-H}$ = 7.2 Hz, NH), 8.12 (t, $^{3}J_{H-H}$ = 5.7 Hz, NH), 8.09 (t, $^{3}J_{H-H}$ = 5.5 Hz, NH), 8.00 – 7.91 (m, 3xNH), 7.81 (s, 1H), 6.42 (s, NH), 6.36 (s, NH), 4.37 (t, $^{3}J_{H-H}$ = 6.2 Hz, 2H), 4.34 – 4.22 (m, 3H), 4.21 – 4.09 (m, 3H), 3.62 (t, $^{3}J_{H-H}$ = 6.6 Hz, 2H), 3.53 – 3.41 (m, 6H), 3.41 – 3.37 (m, 6H), 3.22 – 3.07 (m, 4H), 3.11 (t, $^{3}J_{H-H}$ = 7.4 Hz, 2H), 3.03 (p, $^{3}J_{H-H}$ = 6.5 Hz, 2H), 2.86 (dd, $^{2}J_{H-H}$ = 12.4, $^{3}J_{H-H}$ = 5.1 Hz, 1H), 2.57 (d, $^{2}J_{H-H}$ = 12.4 Hz, 1H), 2.40 – 2.29 (m, 4H), 2.26 (t, $^{3}J_{H-H}$ = 7.9 Hz, 2H), 2.04 (t, $^{3}J_{H-H}$ = 7.4 Hz, 2H), 1.96 – 1.87 (m, 1H), 1.77 – 1.65 (m, 4H), 1.64 – 1.53 (m, 2H), 1.52 – 1.42 (m, 5H), 1.42 – 1.34 (m, 4H), 1.28 (m, 6H); ^{13}C NMR (126 MHz, DMSO-d_6): 174.0 (C), 172.6 (C),

172.5 (C), 171.7 (C), 171.5 (C), 171.4 (C), 170.2 (C), 162.7 (C), 144.6 (C), 122.9 (CH), 70.2 (CH_2), 69.6 (CH_2), 69.4 (CH_2), 69.0 (CH_2), 61.0 (CH), 59.2 (CH), 55.4 (CH), 52.6 (CH), 52.4 (CH), 51.5 (CH), 48.7 (CH_2), 45.4 (CH_2), 42.1 (2xCH_2), 39.8 (CH_2), 38.9 (CH_2), 38.7 (CH_2), 38.6 (CH_2), 35.1 (CH_2), 34.4 (CH_2), 32.0 (CH_2), 31.1 (CH_2), 30.6 (CH_2), 30.5 (CH_2), 30.2 (CH_2), 29.1 (CH_2), 28.6 (CH_2), 28.1 (CH_2), 28.0 (CH_2), 26.9 (CH_2), 26.1 (CH_2), 25.1 (CH_2), 24.9 (CH_2), 22.9 (CH_2); HRMS (ESI, +ve): calcd for $C_{44}H_{72}ClN_{11}O_{11}S_3$ ($[M + H]^+$): 1062.4337, found: 1062.4349.

Compound 26 was prepared following a reported procedure [S3].

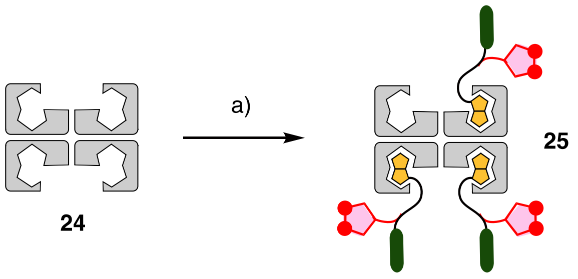

Scheme S1: a) **23**, PBS, rt, 10 min.

Complex 25. To a freshly prepared solution of WT streptavidin (1 mL in PBS, 20 μM), **23** (6 μL, 10 mM in DMSO, 3 equiv) was added and the mixture was shaken for 10 min at rt. Then, the complex **25** was concentrated and washed with PBS (2 × 0.5 mL) using Amicon® Ultra 0.5 mL centrifugal filters (cut off: 30 kDa, 10 min, 14.0 krpm). The material was recovered from the centrifugal filter and diluted again with PBS to the required concentration (i.e., 200 μM, 100 μL).

3 HGM cell line and transient transfection protocol

HeLa cells stably expressing the HaloTag-GFP-Mito fusion protein (HGM): The cells were originally designed by the Chenoweth lab [S4]. They were cultured using the described procedure [S3].

HeLa cells transiently transfected with the GTS-HaloTag-GFP: The plasmid was a gift from the Hensel group [S5]. HeLa cells were seeded at 5×10^4 cells/mL in DMEM + 10% FBS + 1% Pen/Strep on 96-well ibiTreat sterile μ-plates (150 μL per well) and kept at 37 °C with 5% CO_2 overnight. On the next day, transient transfection was performed by diluting the cells with Lipofectamine® 2000–DNA complexes prepared as follows (conditions per well): Lipofectamine® 2000 (0.5 μL) was diluted with Opti-MEM/GlutaMAX reduced serum medium (25 μL) and left to incubate at rt for 5 min. DNA (0.25 μg) was diluted with Opti-MEM/GlutaMAX reduced serum medium (25 μL), gently mixed and added to the diluted Lipofectamine® solution. The resulting solution was gently mixed and incubated for 20 min at rt. The solution of the DNA–Lipofectamine® complex (50 μL each) was added to each well. The cells were kept at 37 °C with 5% CO_2. After 4 h, the medium was exchanged with fresh FluoroBrite DMEM + 10% FBS, and the cells were incubated overnight.

4 HC CAPA optimization

4.1 General HC CAPA protocol

As in [S1]. HGM cells were seeded at 8 × 10^4 cells/mL in FluoroBrite DMEM + 10% FBS on μ-plates 96-well ibiTreat sterile and kept at 37 °C with 5% CO_2 overnight. The next day, the cells were washed with PBS (3 × 3 mL/well) and the media were exchanged to Leibovitz's (4 × 150 μL/well) using a plate washer, keeping a final volume of 135 μL/well. Then, serial dilutions of the corresponding complex **25** in PBS were prepared in a 96-well V-bottom plate and added to the μ-plate containing the cells (15 μL/well, 10 × the final concentration in PBS) to reach a final volume of 150 μL/well (0 to maximum 20 μM). The cells were incubated for 4 h at 37 °C with 5% CO_2. After this, the cells were washed again and **26** was added (15 μL/well, 50 μM in PBS) to reach a final volume of 150 μL/well (5 μM), except for the control wells, where only PBS was added (15 μL/well). After 15 min of incubation at 37 °C with 5% CO_2, the plate was washed again. Then, Hoechst 33342 was added (15 μL/well, 170 μM in PBS) to reach a final volume of 150 μL/well (17 μM). After 15 min of incubation at 37 °C with 5% CO_2, the plate was washed one last time, and the cells were kept in clean Leibovitz's media. During imaging, the samples were kept at 37 °C with 5% CO_2. A total of 16 images/well at 40× were recorded, using three channels: blue (excitation filter: 377/50 nm, emission filter: 477/60 nm, exposure time: 10 ms), green (excitation filter: 475/34 nm, emission filter: 536/40 nm, exposure time: 20 ms) and red (excitation filter: 531/40 nm, emission filter: 593/40 nm, exposure time: 30 ms), as shown in Figure S2. Duplicates were performed for each condition.

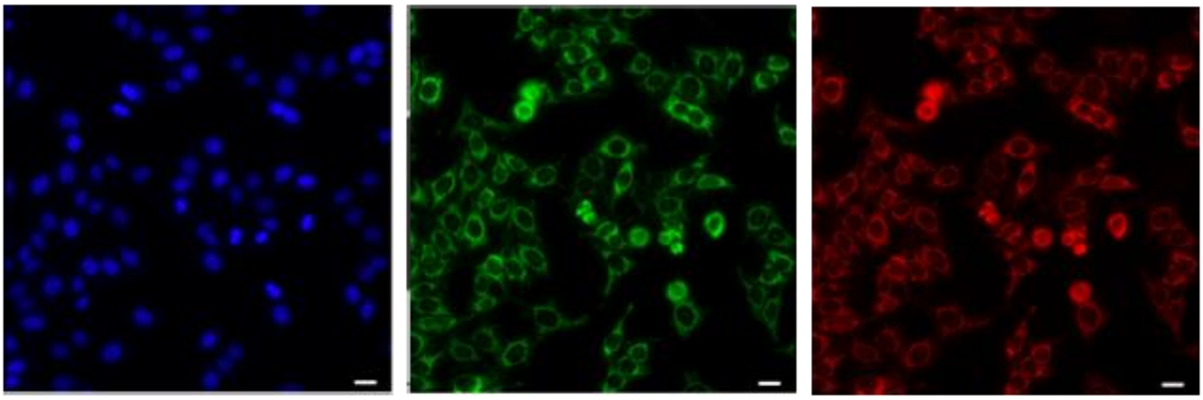

Figure S2: Blue channel recording the Hoechst 33342 localization in the nuclei (left), green channel recording the GFP signal in the mitochondria (middle) and red channel recording the signal from **26** (right) covalently attached to the HaloTag protein in the mitochondria. Scale bar: 10 μm.

4.2 Optimization of the HC CAPA data analysis in HGM cells

HC CAPA was performed as in Section 4.1, except that **25** was not incubated (only **26** and Hoechst 33342 were added). As in [S1], for each cell, the blue and green channel images are used for the segmentation of the nuclei and the whole cell body, respectively (Figure S3).

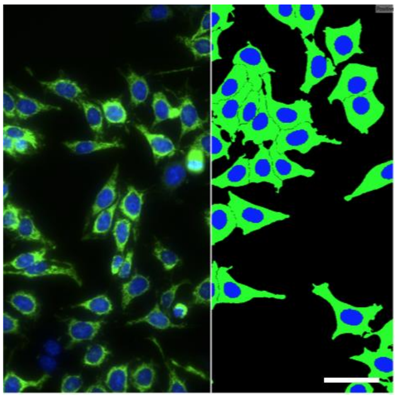

Figure S3: Segmentation of the nuclei (blue, right) and the cell body (green, right) using the green channel (left). Scale bar: 50 μm.

Three successive filters have then been used for the cell selection process (Figure S4):

1) Cells positively expressing GFP (GFP channel with minimum intensity threshold).

2) The shape of the nucleus, to eliminate dead or dividing cell (maximum roundness of 0.7, with roundness = height/length).

3) The size of the nucleus, an additional filter for dividing and dead cells (minimum area of 130 μm^2).

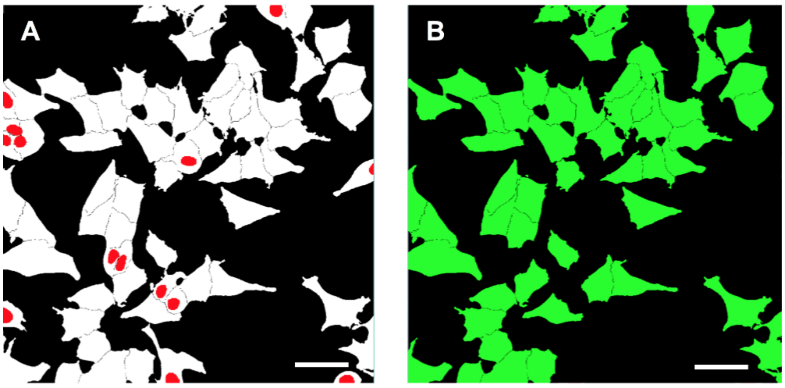

Figure S4: A) Detection of the unregular nuclei (red) for the cell selection. B) Applied cell-body mask after the removal of the dead or dividing cells from the analysis (showing selected cells in green). Scale bar: 50 µm.

Top-hat transform of the green channel image is used to lower the background and to facilitate the segmentation of the mitochondria, creating the corresponding mask (Figure S5).

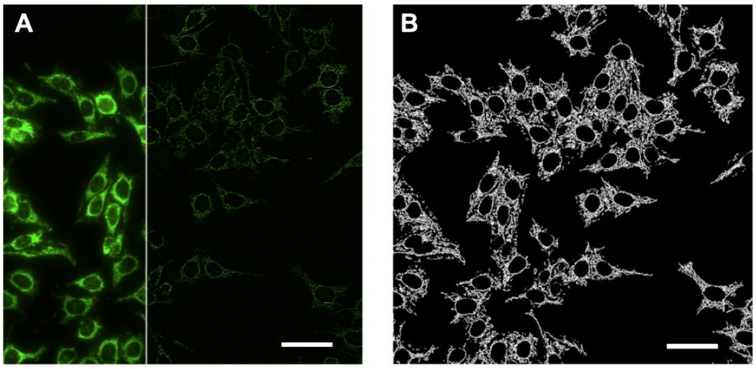

Figure S5: A) Top-hat transform (right) of the green channel image (left). B) Segmentation of the mitochondria using the top-hat transformed image (A, right). Scale bar: 50 μm.

Cell-body and mitochondria mask, in all the cells or only in selected cells, is applied to extract the integrated intensity values (sum of the intensities of the pixels included in the mask) in the red channel image, from the labeling with compound **26**.

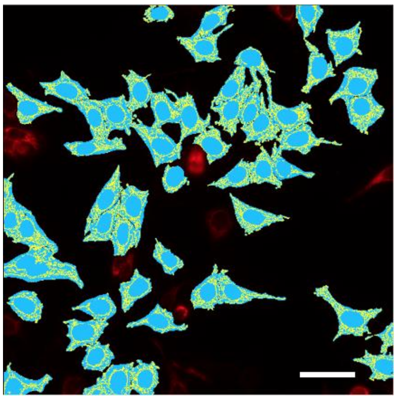

Figure S6: Masks applied for the quantification of the fluorescence intensity of **26**, in the red channel image. Cell body (light blue) and mitochondria (yellow). Scale bar: 50 μm.

To correlate the GFP and **26** fluorescence, integrated intensity sums for each cell (at least 1500 cells) were plotted and fitted with a linear regression to retrieve the goodness of fit (r^2).

4.3 HC CAPA for transient transfection

HeLa cells were transfected as described in Section 3. HC CAPA was performed as in Section 4.1, except that **25** was not incubated (only **26** and Hoechst 33342 were added). As in [S1], during imaging, samples were kept at 37 °C with 5% CO_2. A total of 25 images/well at 40× were recorded, using three channels: blue (excitation filter: 377/50 nm, emission filter: 477/60 nm, exposure time: 20 ms), green (excitation filter: 475/34 nm, emission filter: 536/40 nm, exposure time: 50 ms) and red (excitation filter: 531/40 nm, emission filter: 641/75 nm, exposure time: 15 ms), as shown in Figure S7. Duplicates were performed for each condition.

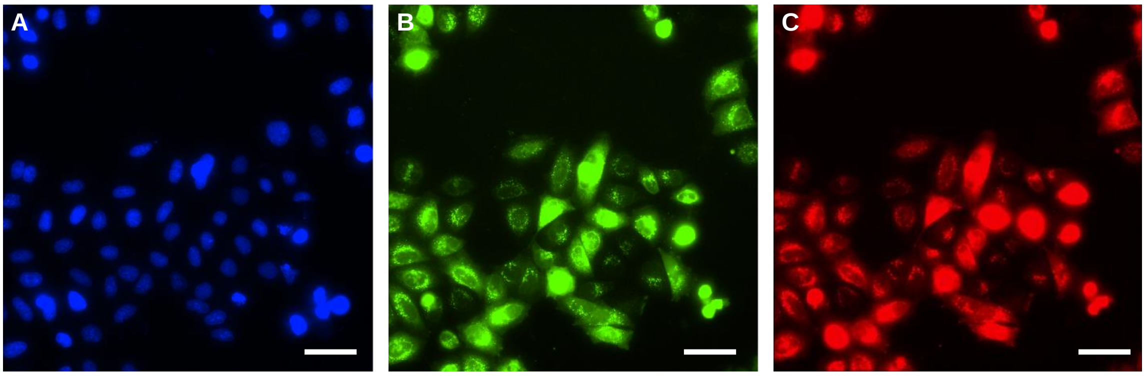

Figure S7: A) Blue channel recording the Hoechst 33342 localization in the nuclei. B) Green channel recording the GFP signal in the mitochondria. C) Red channel recording the signal from **26** covalently attached to the free HaloTag protein in the Golgi apparatus. Scale bar: 50 μm.

4.4 Data analysis of the HC CAPA for transient transfection

For each cell, the blue channel image is used for the segmentation of both nuclei and the whole cell body (Figure S8).

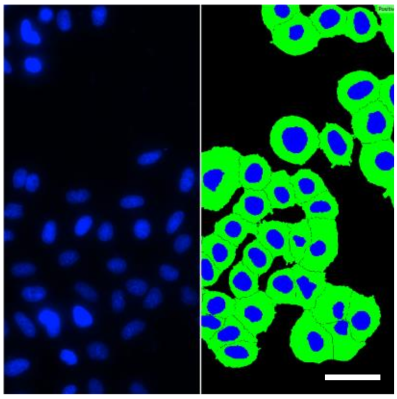

Figure S8: Segmentation of the nuclei (blue, right) and cell body (green, right) using the blue channel (left). Scale bar: 50 μm.

Top-hat transform of the green channel image is used to lower the background and to facilitate the segmentation of the Golgi apparatus, creating the corresponding mask.

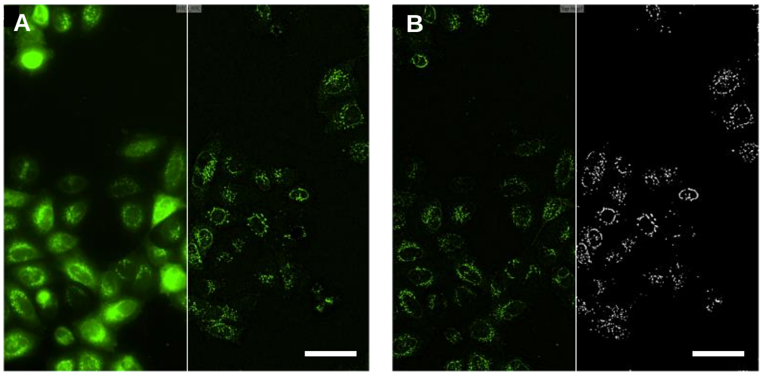

Figure S9: A) Top-hat transform (right) of the green channel image (left). B) Segmentation of the Golgi apparatus (right) using the top-hat transformed image (left). Scale bar: 50 μm.

The area of the Golgi apparatus is expanded and then subtracted from the whole cell body, creating a mask for the cytosolic area (Figure S10).

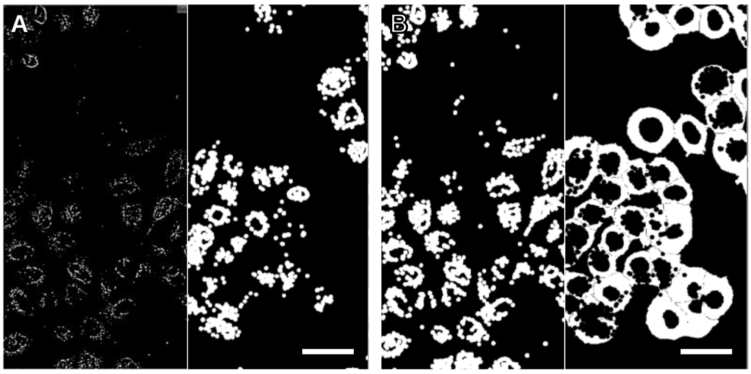

Figure S10: A) Growth of the Golgi apparatus area (right) from the Golgi apparatus mask (left). B) Segmentation of the cytosolic area (right) by subtraction of the enlarged Golgi apparatus to the whole cell body (left). Scale bar: 50 μm.

Cells with low or no GFP signal in the cytosolic area are then differentiated from the overtransfected cells, with a high GFP signal in the whole cell body (Figure S11). Cell body and Golgi apparatus masks, in all the cells or only in selected cells, are applied to extract the integrated intensity values (sum of the intensities of the pixels included in the mask) in the red channel image, from the labeling with compound **26**.

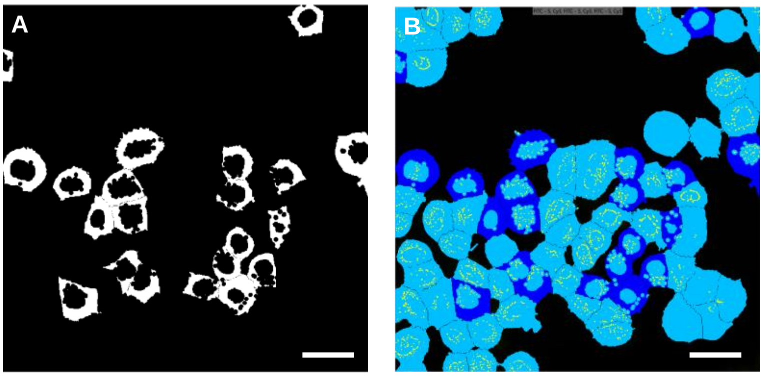

Figure S11: A) Mask for cytosolic areas with low or no GFP signal. B) Applied cell body mask (light blue), Golgi mask (yellow), and mask of cell regions with low or no GFP signal (dark blue, mask in A) in all the cells. Scale bar: 50 μm.

To correlate the GFP and **26** fluorescence, the integrated intensity sums for each cell (at least 1500 cells) were plotted and fitted with a linear regression to retrieve the goodness of fit (r^2).

4.5 HC CAPA results for the uptake quantification

HC CAPA was performed as in Section 4.1. and the data analysis as in Section 4.2. The integrated intensity values for each condition using optimized HC CAPA were then normalized using the value of the integrated intensity with the addition of **26** (0 µM of **25**) as a maximum signal (I_{rel} = 1) and the value of the integrated intensity without the addition of **26** (0 µM of **25**) as a minimum (I_{rel} = 0), for each set of experiments. Duplicates were performed for each condition.

The resulting dependence of the relative intensity values (I_{rel}) from the concentration of **25** ($c_{\mathbf{25}}$) was plotted and fitted with Equation S1 to retrieve the half maximal effective concentration (CP_{50}) value (n is the Hill coefficient).

$$I_{rel} = 1 / (1 + (c_{\mathbf{25}} / CP_{50})^n) \qquad (S1)$$

The cytotoxicity was evaluated by calculating the ratio between the number of selected and nonselected cells and fitted with a linear regression.

5 References

[S1] López-Andarias, J.; Saarbach, J.; Moreau, D.; Cheng, Y.; Derivery, E.; Laurent, Q.; González- Gaitán, M.; Winssinger, N.; Sakai, N.; Matile, S. *J. Am. Chem. Soc.* **2020**, *142*, 4784–4792. doi.org/10.1021/jacs.9b13621.

[S2] Martinent, R.; Du, D.; López-Andarias, J.; Sakai, N.; Matile, S. *manuscript in preparation*.

[S3] Peraro, L.; Deprey, K. L.; Moser, M. K.; Zou, Z.; Ball, H. L.; Levine, B.; Kritzer, J. A. *J. Am. Chem. Soc.* **2018**, *140*, 11360–11369. doi:10.1021/jacs.8b06144.

[S4] Ballister, E. R.; Aonbangkhen, C.; Mayo, A. M.; Lampson, M. A.; Chenoweth, D. M. *Nat. Commun.* **2014**, *5*, 5475. doi.org/10.1038/ncomms6475.

[S5] Liss, V.; Barlag, B.; Nietschke, M.; Hensel, M. *Sci. Rep.* **2015**, *5*, 17740. doi.org/10.1038/srep17740.

The original data can be found at:

http://doi.org/10.5281/zenodo.3925076

6 NMR spectra

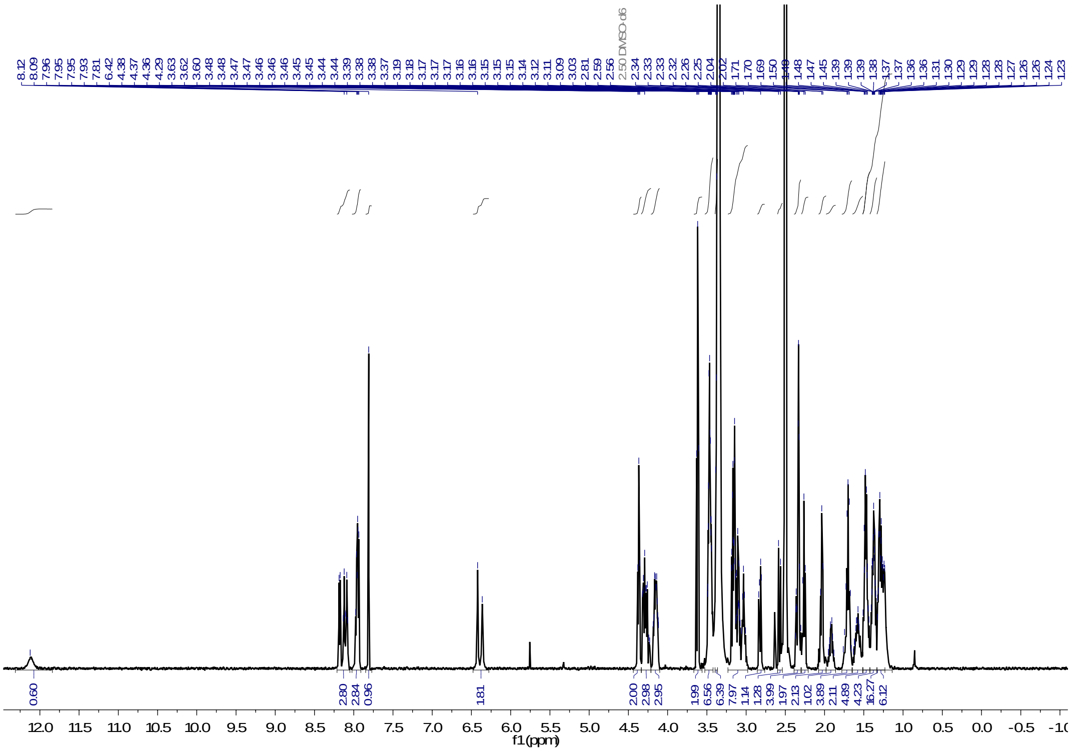

Figure S12: ^{1}H NMR (500 MHz, DMSO-d_6) spectrum of **23**.

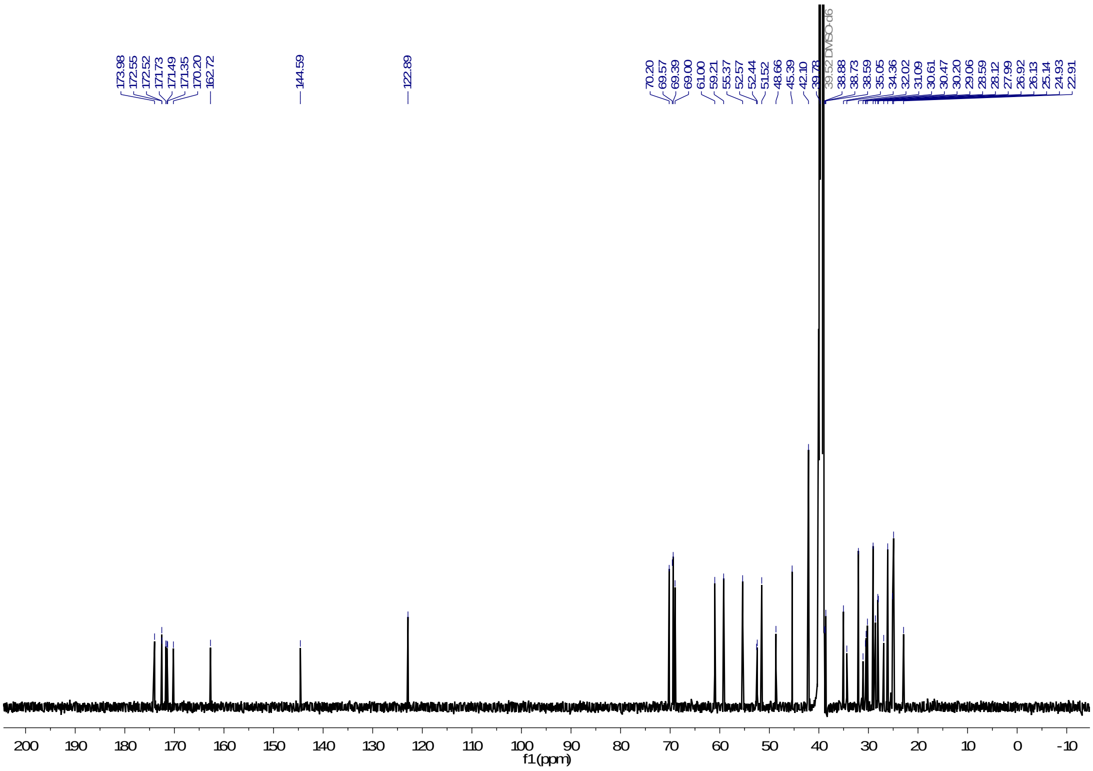

Figure S13: ^{13}C NMR (126 MHz, DMSO-d_6) spectrum of **23**.

7 LC–MS spectra

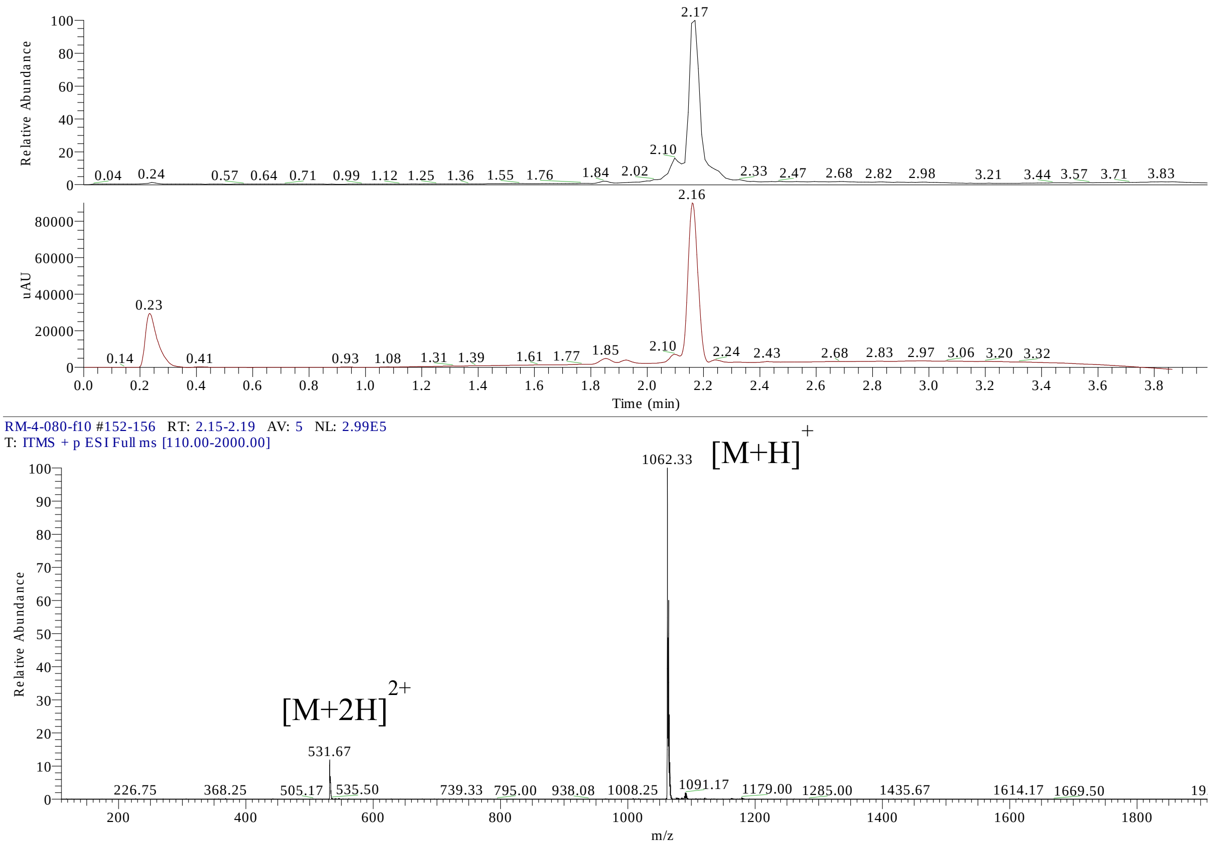

Figure S14: LC–MS (ESI$^+$) trace of **23**.
